# Update on the Pathophysiology and Management of Tics

**DOI:** 10.1007/s11910-026-01480-8

**Published:** 2026-03-04

**Authors:** Emily Casaletto, Lucy Morse, Darren Miller, Juan Deliz-Gonzalez, Danielle Larson

**Affiliations:** 1 Department of Neurology, Northwestern University Feinberg School of Medicine, Chicago, United States; 2 Department of Psychiatry, Northwestern University Feinberg School of Medicine, Chicago, United States; 3 Abbott Hall 1115, 710 N Lake Shore Dr., Chicago, IL 60657 United States

**Keywords:** Tourette syndrome, Tic disorders, Functional tic like behaviors functional neurologic disorder, OCD, ADD

## Abstract

**Purpose of Review:**

This review aims to collate takeaways from the most recent and relevant literature related to tics, from genetic studies to case studies elucidating Functional tic like behaviors (FTLBs) and clinical trials of novel drugs in development.

**Recent Findings:**

Recent genome-wide association studies (GWAS) and functional neuroimaging studies have enhanced the understanding of genetic and structural links to Tourette Syndrome (TS). The rise of FTLBs during the Covid-19 pandemic heightened our understanding of this phenomenon and led to the identification of social media’s influence on tics. New studies have identified sex-related difference in TS and common psychiatric co-morbidities. Tic treatment is evolving away from traditional anti-psychotics toward newer compounds including VMAT-2 inhibitors, Ecopipam, and cannabinoid formulations, as well as novel transcranial stimulation approaches.

**Summary:**

Our understanding of tic etiology and pathophysiology as well tics’ functional counterpart FTLBs and social media impact is expanding along with our ability to manage tics with novel treatments in development.

## Introduction

Though it is a complicated neuropsychiatric entity, our understanding of Tourette Syndrome (TS) steadily improved since it was first described in 1885 by Georges Gilles de la Tourette. TS is characterized by the presence of both motor and phonic tics with onset prior to age 18, with tics described as sudden, brief, stereotyped, temporarily suppressible and purposeless movements with an associated premonitory urge and subsequent relief [1]. Aspects of TS, from its underlying etiology and pathophysiology to its relation to functional mimicry and psychiatric comorbidities are still being discovered and better characterized. The phenomenon of actions mimicking tics has evolved into well-defined FTLBs due to their recently increased prevalence, catalyzed by both the Covid-19 pandemic and the exponential ubiquity of social media. Just as our understanding of TS and its related disorders and phenomena are growing, so too is our ability to treat tics beyond the standard anti-psychotic pharmacology as anti-dopaminergic compounds, cannabinoids, and non-pharmacologic treatments are being developed and investigated. This review provides an overview of these various topics currently shaping our understanding of tics and TS through examination of recent, relevant literature. The authors have no conflicts of interest to declare regarding the content of this article.

## New Understandings in Tic Pathophysiology

While the exact underlying cause and mechanism of Tourette Syndrome and other tic disorders has not been fully elucidated, advances in genetics research and functional neuroimaging and have shed additional light in recent years. While the heritable nature of TS has been well understood, with the presumption of a polygenic underpinning, a large genome-wide association study (GWAS) published in 2019 showed that of nearly 5,000 individuals with TS, genetic variants of evolutionarily-conserved regions explained 92.4% of TS heritability (p-value = 0.005) [2]. Similarly leveraging knowledge from the latest GWAS, inclusive of toughly 93,000 individuals, Yang et al. identifying multiple novel genetic regions and hits playing a pleiotropic role across TS, ADHD, ASD, and OCD – disorders that are known to be linked clinically [3].

Complementing the advances in genetic underpinnings of TS, advances in neuroimaging studies have further elucidated the role of the prefrontal cortex in TS, adding to our understanding of tic pathophysiology. Tics, and thereby TS, have been linked to aberrancy in the cortico-striatal-thalamic-cortical (mesolimbic) circuit with resultant motor cortex and limbic system disinhibition and hyper-reactivity [4]. Adding to this picture is the understanding that the prefrontal cortex is also hyperactive in individuals with tics, ostensibly to overcome greater motor drive from basal ganglia and primary motor cortex. Using fMRI, Rae at al. found the inferior frontal gyrus – in addition to other areas including the pre-SMA and insula – to have elevated activity during voluntary and reactive inhibitions in individuals with TS [5]. Yu et al.’s large GWAS findings adds credibility to unique pre-frontal activity in tics through the elucidation that TS-associated genes were preferentially expression in human dorsolateral prefrontal cortex [2].

## Functional Tic Like Behaviors: Where Are They Now?

### Emergence of FTLBs

Functional tic-like behaviors (FTLBs) have long been recognized as a manifestation of functional movement disorders, though they have only been formally defined recently in the setting of rising prevalence. FTLBs are involuntary movements or vocalizations that mimic those seen in tic disorders without an “organic” neurologic etiology [6]. Historically, the prevalence of FTLBs has been as low as less than 5% of patients who present with FMD [7, 8]. Prior to the year 2020, patients with FTLB were primarily described in the literature through small case series [9–11]. However, by the summer of 2020, notably after the start of the Covid-19 pandemic, there was a dramatic rise in FMD and FTLB presentations globally [12, 13]. One single-center retrospective cohort study of 185 individuals referred to a pediatric tic clinic noted children noted FTLB prevalence of 36% in 2021 compared to 2% in 2018 [14]. Another multi-center tic disorders clinical registry saw FTLB referrals jump from between 1 and 5% prior to 2021 to 20–35% in June 2021 [15]. Factors that contributed to this increase in FTLBs are hypothesized to include an increase in awareness and ability to diagnose FMD, psychosocial stressors (e.g., social isolation, financial impact of Covid-19 pandemic), heightened anxiety, and exposure to tics and tic-like behaviors on social media [15–17].

### FLTB Clinical Presentation

**Fig. 1:**
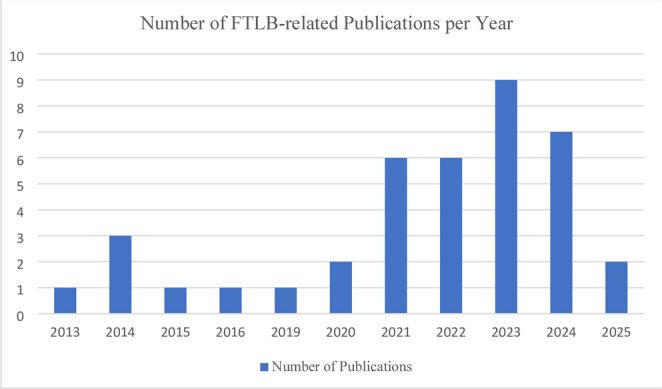
This graph demonstrates the trend of increasing publications related to FTLBs since the Covid-19 Pandemic began in 2020. It suggests there may be a more recent tapering of publications, though the remainder of 2025 is undetermined. Publications are those included in this review, identified in our search of the literature

The increased prevalence of FTLBs enable movement disorders specialist to further characterize them, expanding on previously limited knowledge, as a 2019 review of FTLBs noted a challenge indistinguishing between the two entities, which can coexist, commonly leading to misdiagnosis due to the dearth of available literature [18]. In the wake of the Covid-19 pandemic, there has been an increase in studies and publications on FTLBs (Fig. 1), which have consistently identified differences between TS and FTLB across domains including demographics, risk factors, phenomenology, and clinical course. Key differences between these tic disorder conditions are outlined in Table 1. Compared to TD, which is most often seen in males during early childhood, patients with FTLB tend to be female or gender diverse and onset tends to occur during adolescence or young adulthood [15, 19–23]. Additionally, patients with TD tend to have a strong family history of tics, which is absent in most individuals with FTLB [19]. Regarding psychiatric co-morbidities. Like TS, patients with FTLB also have higher rates OCD, ADHD and ASD compared to the general population, but compared to patient with TS they have higher rates of depression, anxiety, and family members with psychiatric disorders [14, 18, 20, 21, 23, 24]. Notably, patients were more likely to have experienced an adverse psychosocial event shortly before symptom onset [19, 25].

**Table 1.** Key differences distinguishing FTLBs and classic tic disorders

	FTLBs	Classic Tic Disorders (i.e. Tourette Syndrome)
Phenomenology	Complex movements Self-injurious behaviors Coprolalia Repetition of long phrases	Simple, brief, jerk-like movements (i.e. head movement, shoulder shrug, eye blink) Brief vocalizations (i.e. throat clearing, exhale)
Age of Onset	20–30 s	6–9
Sex / Gender	Female Non-binary	Male
Common Co-morbidities	Anxiety Depression Functional Neurologic Disorder (FND) Psychogenic Non-Epileptic Spells (PNES)	ADD/ADHD OCD Autism Spectrum Disorders
Treatment	Treatment of psychiatric co-morbidities Psychotherapy for FND/PNES CBIT/HRT for FTLBs	

### FTLB Phenomenology

As is often the case in neurology, perhaps the most useful tool in differentiating the two entities is the physical exam. Whereas organic tic disorders typically presents as a simple motor (most commonly in the face) or vocal tic, FTLB presents as complex movements and vocalizations. These atypical movements seen in FTLB commonly involve the trunk and extremities, are large in amplitude, and can be self-injurious to the patient [19, 20, 25, 50]. Similarly, vocalizations frequently include complex phrases and coprolalia-like behaviors, including clicking, whistling, and a range of odd, obscene, or offensive phrases [14, 20, 51]. While both patients with TD and FTLB report premonitory urges that are similar in frequency and intensity, descriptions of these sensations tend to differ [52]. A large retrospective review found that TS patients me described premonitory urges as brief sensations of tension or itchiness usually in the body region where the tic later occurs, whereas patients with FTLB described sensations such as “electric shocks” or “tongue vibration” often involving the entire body or in regions separate from where the tic occurs [25]. Features classically seen in functional movement disorders, including distractibility and suggestibility, are common in FTLBs [12].

### FTLB Clinical Course

The clinical course of TD and FTLB diverges as well. In TD, onset is usually insidious, beginning with simple motor tics that wax and wane in frequency and severity, and can increase in complexity over a period of years. This progression is typically retro-caudal [18]. On the contrary, patients with FTLB often report rapid symptom inset over a period of hours to days that peaks within one month [20–22, 50]. There is less symptom fluctuation though symptoms do tend to deteriorate in the presence of others [50]. In terms of functioning, patients report lower quality of life, greater disability and absenteeism, and more hospitalizations [14, 53]. In a retrospective review of 56 patients with new-onset FTLB after March 2020, 79% of patients improved independent of treatment [23].

### FTLB Management

Unsurprisingly, most patients with FTLB experience no benefit with typical pharmacologic treatment for TD [20, 23, 25]. As with other functional movement disorders, a multidisciplinary approach including education, addressing comorbidities, and cognitive behavioral therapies (CBT) is likely essential, though an optimized approach has not been standardized [54]. Education remains the cornerstone of initial management; evaluation of an online psychoeducation group for patients with FTLB showed significant improvement in patient-reported outcomes [55]. Multiple studies have also shown treatment with SSRIs and CBT to be effective [56–58]. More prospective studies are needed to evaluate effective treatment of FTLB.

### FTLB Diagnostic Criteria

The identification of common features of FTLB was monumental in identifying positive diagnostic criteria and shifting the diagnosis from a diagnosis of exclusion to one of inclusion. In October 2022, twenty-four tic specialists, many of whom authored of the aforementioned studies, developed the European Society for the Study of Tourette Syndrome (ESTSS) 2022 criteria for clinical diagnosis FTLB [81]. The criteria include three major criteria and two minor criteria, which has preliminarily shown utility both in diagnosing individuals with exclusively FTLB as well as patients with Tourette syndrome and functional overlay [21, 59, 60].

The surge in FTLB presentations that was clearly documented between 2020 and 2022 appears to be declining. A large single-center cohort that evaluated patients who developed FTLB between April 2020 and March 2023 showed a peak in prevalence in 2022 [61]. There are fewer reports documenting the prevalence of FTLB since the height of the Covid-19 pandemic, however there does appear to be a persistence of new FTLB diagnoses [22, 51, 62]. Additional studies beyond 2023, specifically longitudinal population-based studies, would be helpful in fully mapping the trajectory of FTLB and long-term impact of the increase seen during the Covid-19 pandemic.

## Social Media Influence on Tics and FTLBs

Though the rise of FTLBs in 2020 was spurred in large part by the Covid-19 pandemic, an important instigating factor was the influence of social media, which has persisted. This was recognized early in a tic-based clinic registry capturing 20 cases of FTLBs presenting in 2020 that all endorsed social media exposure to content creators with tics [21]. The phenomenology of FTLBs has been linked to mimicry of video-based content creators, with One early case series documented 6 teenage girls with similar, explosive onset tic-like behaviors, lacking suppressibility, and often triggered by particular external stimuli, all reporting exposure to one particular British Tik Tok content creator prior to symptom onset, and some displayed specific movements and sounds shared by that creator [63].

Recognition of this surge in FLTBs, and in particular with features shared by key high-view videos on Tik Tok and other social media platforms, has been characterized by some as part of a phenomenon known as ‘mass sociogenic illness’-- and while this concept has been previously described in groups with shared physical social space or geography, the framework has been expanded to include those accessing shared social media resources and communities, and has been specifically coined as ‘mass social media-induced illness’ [25]. In the case of TikTok specifically, the phenomenon of ‘TikTok Tics’-- specifically, those movements self-labeled as tics or Tourette Syndrome-related by content creators themselves – has developed. This phenomenon was thoroughly explored and well documented by Olvera et al. in their study of TikTok videos associated with the hashtag label of ‘tic’, ‘Tourette’, or ‘tourettes’ in which they found the phenomenology to resemble FTLBs with high percentage of large-amplitude arm movements, a paucity of simple, mild, or infrequent tics, a high incidence of coprolalia and copropraxia as well as a high incidence of self injurious behavior [64]. A similar investigation assessed the 100 most viewed videos labeled under the hashtag ‘tourettes’, and reported high incidence of coprophenomena, strong environmental triggers, and injurious or aggressive behaviors that were overall rated by investigators to be atypical of Tourette Syndrome [65].While these findings clearly represent the predominance of FTLBs in the social media landscape more work needs to be done, from a clinical perspective, to understand the influence of social media on organic tic disorders.

## Considering of Psychiatric Comorbidities

The link between TS and psychiatric co-morbidities has long been recognized, with one of the more robust studies of the prevalence indicating that 85.7% of individuals living with TS are diagnosed with a psychiatric illness in their lifetime, 57.7% with two, most commonly OCD, ADHD or ASD [66]. A developing nuanced understanding of this neuropsychiatric overlap is the identification of sex differences whereby male sex is associated with higher diagnoses rates of TS and ASD, but female sex is associated with greater progression of symptom burden into adulthood, raising the question of causality between biological difference in symptom expression or social and educational factors [67, 68]. Further research is needed to elucidate key differences in tic disorder experience across sex and gender, as any differences could have care management implications.

Regarding recommended treatment of co-morbid OCD, a recent systematic review and meta-analysis demonstrated that antidepressants effectively alleviate symptoms for nearly one third of patients, and combination with an antipsychotic, commonly aripiprazole or risperidone, is effective for two thirds of remaining individuals [69]. For the treatment of comorbid TD and ADHD, methylphenidate, atomoxetine, or clonidine (or a combination thereof) have the strongest evidence; studies also suggest possible benefit from desipramine, guanfacine, clonidine, selegiline, and dextroamphetamine [70]. Contrary to previous guidelines, these medications for TD and ADHD generally do not worsen tics, with a notable exception of dextroamphetamine at higher doses [70].

## Emerging Therapies for Tic Disorders

The management of tic disorders is evolving beyond traditional dopamine receptor antagonists due to concerns over systemic side effects and limited efficacy. Recent advances include novel pharmacotherapies and interventional approaches that offer targeted treatment with improved tolerability, as outlined in Table 2.

**Table 2.** Treatments in development for tic management

Drug/Treatment	Summary and Findings from Clinical Trials	References
Ecopipam	❖ A selective dopamine D1 receptor antagonist ❖ A 12 week trial showed significantly reduced Yale Global Tic Severity Scale (YGTSS) scores in pediatric TS patients compared to placebo with mild adverse effects	Cheng et al. [26] Gilbert et al. [27] Gilbert et al. [28]
VMAT2 Inhibitors (e.g., Deutetrabenazine)	❖ Open-label studies showed promise for deutetrabenazine ❖ ARTISTS trials found no significant benefit in tic severity despite some exploratory numeric improvements ❖ Valbenazine also showed no significant efficacy in reducing tics	Jankovic et al. [29] Jankovic et al. [30] Coffey et al. [31] Jankovic et al. [32] Farber et al.[33]
Δ9-Tetrahydrocannabinol (THC)	❖ Small trials suggest THC may reduce premonitory urges and tic frequency/severity ❖ Larger studies have inconsistent results ❖ A 6-week crossover trial with THC/CBD oil showed a significant reduction in tic severity, with concerns over cognitive side effects and small sample size	Szejko et al. [34], 16. Serag et al. [35]
Deep Brain Stimulation (DBS)	❖ Considered for severe, treatment-refractory cases ❖ More invasive treatment options ❖ Limited long-term data, particularly in younger populations	Aydin et al. [36] Wehmeyer et al. [37] Zhang et al. [38] Krack et al. [39]
Repetitive Transcranial Magnetic Stimulation (rTMS)	❖ No statistically significant improvement in tic severity ❖ Moderate reductions in premonitory urges and OCD symptoms ❖ Current trials are investigating targeted modulation of the supplementary motor area	Dyke et al. [40] Godiero et al. [41] Kleimaker et al. [42] Aloufi et al. [43] Steuber et al. [44] Lin et al. [45]
Transcranial Direct Current Stimulation (tDCS)	❖ A small trial suggested that cathodal tDCS over the bilateral SMA may reduce motor tic severity, showing preliminary promise	Conelea et al. [46] Kahl et al. [47] Landeros-Weisenberge et al. [48] Mahjoub et al. [49]

This table provides the highlights from the most recent clinical trials of various drugs and non-pharmacologic interventions in development for improving the treatment of tics, with relevant references listed.

Second-generation antipsychotics with improved side effect profiles have largely supplanted older dopamine receptor antagonists in the treatment of tics. Aripiprazole, a partial D2 receptor agonist, has emerged as a first-line pharmacological option due to its favorable tolerability, including reduced risk of weight gain and extrapyramidal symptoms compared to haloperidol or risperidone [71]. Most studies on the use of aripiprazole for tics have focused on the pediatric and adolescent populations, leading to a relative lack of robust evidence for its use in adults [72, 73]. Other newer agents, such as lurasidone and brexpiprazole, are being explored off-label, though data remain limited [26, 74, 75].

Ecopipam, a selective dopamine D1 receptor antagonist, has shown promise in reducing tic severity without the extrapyramidal side effects commonly associated with D2-receptor blocking agents [76]. In a randomized, double-blind phase 2b, 12-week trial, ecopipam significantly reduced Yale Global Tic Severity Scale (YGTSS) scores in pediatric Tourette syndrome (TS) patients compared to placebo [27]. Adverse effects were generally mild, with headache, insomnia, fatigue and somnolence being most common. Furthermore, ecopipam continued to be well-tolerated without new adverse events over 12 months in an open-label extension phase of the same study [28].

VMAT2 inhibitors, which are FDA-approved for other hyperkinetic conditions and act by depleting presynaptic dopamine, have generally not demonstrated efficacy in the treatment of tics in clinical trials. Although tetrabenazine has long been used off-label, newer agents have been more closely investigated for TS. In an open-label 8-week study, deutetrabenazine initially showed promise in safely reducing tic severity in adolescents with TS) [29]. More recently nonetheless, the ARTISTS 1 (flexible dosing) and 2 (fixed dosing) trials evaluated the efficacy and safety of deutetrabenazine in treating tics associated with TS [30, 31]. Despite employing differing dosing strategies, both studies failed to meet their primary efficacy endpoints of reduction in tic severity. Similarly, a 54-week open-label extension of the ARTISTS trials did not demonstrate a statistically significant benefit in reducing tics, though exploratory analyses indicated some numeric improvements in tic severity and quality of life [32]. A series of six studies (including randomized controlled trials and open-label extension studies) of valbenazine also did not demonstrate significant efficacy in reducing tics in TS patients [33].

Cannabinoid compounds, particularly Δ9-tetrahydrocannabinol (THC), have gained interest for tic suppression in TS [34]. Small randomized controlled trials and observational studies suggest that THC may reduce premonitory urges and tic frequency and severity [35]. Nabiximols, a standardized THC: CBD oromucosal spray, has shown early promise in open-label trials but has not yet demonstrated consistent efficacy in larger studies [77]. A double-blind, randomized, 6-week crossover trial evaluated the efficacy of a 1:1 THC/CBD oral oil in twenty-two adults with severe TS [78]. Results showed a significant reduction in tic severity during active treatment compared to placebo, in addition to improvements in obsessive-compulsive symptoms and anxiety. Notably, adverse effects while on active treatment included cognitive difficulties such as slowed mentation, memory lapses and poor concentration in some participants, raising concern for tolerability (particularly in younger individuals). The study was also limited by a small sample size and short treatment period. Other recent randomized controlled studies exploring differing compositions of medical cannabis have also been limited by low sample size and high discontinuation rates [79, 80].

Deep brain stimulation (DBS) may be considered for severe, treatment-refractory cases [36]. Common targets include the centromedian-parafascicular (CM-Pf) thalamic complex and the globus pallidus internus (GPi) [37, 38]. Technological innovations like adaptive (“closed-loop”) DBS are under development to optimize stimulation based on real-time neural feedback [39]. While promising, DBS remains an invasive option requiring multidisciplinary assessment, and long-term data, especially in younger populations, are limited.

An exciting area of current and future research is in device-related advanced therapies for TS. Repetitive transcranial magnetic stimulation (rTMS) and transcranial direct current stimulation (tDCS) are emerging non-invasive neuromodulation techniques under investigation for TS [40, 41]. These approaches aim to modulate cortical excitability and connectivity, particularly in motor and prefrontal circuits implicated in tic generation [42]. Recent meta-analyses have not supported a statistically significant improvement in tic severity with rTMS [43, 44]. Nonetheless, a moderate reduction in premonitory urge and OCD symptoms have been observed with rTMS [45]. Two randomized, sham-controlled trial are underway looking into targeted modulation of the supplementary motor area (SMA) using TMS to potentially augment the efficacy of comprehensive behavioral intervention for tics (CBIT) in young individuals with chronic tics [46, 47]. A prior trial had failed to demonstrate efficacy of 3-week SMA-targeted TMS monotherapy in adults [48]. Finally, a recent small (*N* = 24) randomized, sham-controlled trial provided preliminary evidence that cathodal tDCS over the bilateral SMA may reduce motor tic severity [49].

## Conclusions

While there are still unknowns related to tic etiology, related phenomenon, and treatment capacity, we have come a long way in recent years, starting with more data-driven pathophysiologic theories backed by breakthroughs in studies of genetics and functional neuroimaging. We have now defined tic-mimicry into a known entity, FTLB, enabling accurate diagnosis and management, and better understand the influential forces of social media. Complementing our advanced understand of tics and their related conditions, soon we may have novel treatments for tics to minimize our use of anti-psychotics and provide more individualized patient care.

## Key References

- Pringsheim T and Martino D. Rapid onset of functional tic-like behaviours in young adults during the COVID-19 pandemic. Eur J Neurol 2021:28:3805-3808. 20210804. DOI:10.1111/ene.15034.
  - Among the first papers to describe the higher incidence in functional tic mimicry, and use the term FTLBs, observed during the Covid-19 pandemic, paving the way for others to further describe this new clinical entity.
- Olvera, C, Stebbins G, Goetz C, Kompoliti K. TikTok tics: a pandemic within a pandemic. Mov Dis Clin Prac 2021:8:1200-1205. 
  - An informative deep-dive on social media’s influence on tics and FTLBs, providing more clarity on this societal phenomenon.
- Panda PK, Panda P, Dawman L, Mishra AS, Kumar V, & Sharawat IK. (2025). Safety and Efficacy of Ecopipam in Patients with Tourette Syndrome: A Systematic Review and Meta-analysis CNS drugs 2025:39(2):127–142.
  - A recent review of Ecopipam’s safety and efficacy – arguably the first tic-specific drug to be developed and studied.
- Mahjoub Y, Szejko N, Gan LS, et al. Randomized Controlled Trial of Transcranial Direct Current Stimulation over the Supplementary Motor Area in Tourette Syndrome. Mov Disord Clin Pract. 2025:12(3):313-324. doi:10.1002/mdc3.14285.
  - Results of a recent RCT of an emerging non-pharmacologic therapy for tics.

## Data Availability

No datasets were generated or analysed during the current study.
